# Paddle selection and adjustment for young male kayakers: the science behind the art

**DOI:** 10.3389/fphys.2025.1713935

**Published:** 2025-12-10

**Authors:** Rui António Fernandes, Fernando Alacid, Beatriz Branquinho Gomes

**Affiliations:** 1 Faculty of Sport Sciences and Physical Education, CIPER (Interdisciplinary Center for the Study of Human Performance), University of Coimbra, Coimbra, Portugal; 2 Faculty of Education Sciences, Health Research Center, University of Almeria, Almeria, Spain; 3 CEMMPRE (Centre for Mechanical Engineering, Materials and Processes), University of Coimbra, Coimbra, Portugal

**Keywords:** kayaking, paddle setup, maturity, youth, anthropometry

## Abstract

This study aimed to produce predictive equations to determine the optimal paddle size for young Iberian kayakers competing in three categories: U14, U16, and U18. Data were collected in Portugal and Spain between 2005 and 2018, with a sample that includes 149 kayakers aged 14.42 ± 1.20 years. The kayakers were assessed for somatic maturation using the maturity offset and the percentage of the predicted adult height (%PAH). For anthropometry, assessing body mass (kg), stretch stature (cm), sitting height (cm), arm span (cm), arm length (cm), forearm length (cm), biacromial diameter (cm) and chest girth (cm), and for equipment setup, measuring paddle and blade length, blade maximum width, handgrip distance. A stepwise multiple linear regression analysis was performed to determine which anthropometric and maturity attributes could predict the paddle configuration for the total sample and each category analysed. Significant differences were found between the three categories for anthropometry, paddle setup, and maturity, except for PAH. Significant differences between U16 and U18 were only found in CA, maturity offset, %PAH, and paddle length. The predictive equation for the total sample explains the variance in paddle length by 75% and may be used as a more objective guide for paddle scaling.

## 1 Introduction

Optimising sporting equipment is crucial for reaching peak performance in competitive sport and to ensuring athlete comfort and injury prevention. However, sports equipment design is often constrained not by the natural laws of physics but by the rules of the sport (Shan, 2008).

Canoeing is a widely practiced sport worldwide and has been an Olympic event since the 1936 Berlin Games. In sprint kayaking, a defined sequence of cyclical movements is repeated (Kukić et al., 2022), using a double-blade paddle to propel the kayak.

According to the competition rules for canoe sprint of the International Canoe Federation, published in January 2025, the paddles must not be attached to the boat in any way. No other regulations regarding the shape and size of the paddle and its respective blades are suggested. In this discipline, young kayakers use the same materials, often with the same characteristics as adult athletes.

One of the most challenging tasks for coaches is accurately selecting the correct paddle size. The paddle is typically sized based on the athlete’s anthropometry, paddling style, technique, strength, and other factors. Empirically, the most common method for determining paddle length is to stand it vertically alongside the kayaker, with the arm stretched. If the fingers curl over the top blade, the paddle is considered about the right size. However, this method is not ideal and often yields longer-than-necessary paddles. For determining the correct hand placement, the most commonly used method involves holding the paddle above the head with the arms stretched out horizontally, the forearms forming a right angle, and dividing the paddle into three equal lengths (Rademaker, 1977).

Burke and Pruitt (2003) stated that incorrect equipment adjustments could likely affect the athlete’s comfort, his ability to perform the technical movement under ideal conditions, and, subsequently, his performance. Timmerman et al. (2015) suggested that properly scaling net heights, racquet length, or court size may lead to a more engaging learning environment. Additionally, when combining equipment size with the practitioner’s developmental stage, skills are performed more successfully and with more desirable movement patterns (Buszard et al., 2014; Farrow and Reid, 2010).

In kayaking, the propulsive power generated in a paddle stroke depends on the speed of the blade, and if the paddler’s blade size is greater or smaller than the optimal size, then the energy expended by the kayaker to maintain the race pace would be expected to increase (Sprigings et al., 2006). Which, with an incorrect paddle selection, may lead to injury. It is known that the high physical demands of executing the sprint kayak stroke efficiently may lead to shoulder injuries (Toohey et al., 2019).

Despite that, there is limited normative data on optimising the equipment setup based on human morphology in sprint kayaking (Diafas et al., 2012; Ong et al., 2006; Ong et al., 2005), and even fewer studies have focused on young kayakers (Alacid et al., 2014). Consequently, athletes and coaches in kayak competitions face various equipment setup decisions that impact performance. Often, this tuning process requires hours of practice and relies on the athlete’s subjective feedback, leading to a trial-and-error approach. However, for many athletes, equipment is chosen more for comfort than for any mechanical advantage it may provide (Ong et al., 2005).

Therefore, to reduce the hours of trial and error needed for paddle adjustment, this study aimed to develop predictive equations for determining the ideal kayak paddle size by evaluating the maturational, anthropometric, and current paddle configuration characteristics of young Iberian athletes from three different age groups, Under 14 (U14), Under 16 (U16), and Under 18 (U18).

## 2 Materials and methods

### 2.1 Participants and data collection

Data were collected in Portugal and Spain between 2005 and 2018, with a sample that includes 149 kayakers aged 14.42 ± 1.20 years, ranging from 12.06 to 16.80 years, and distributed over three competitive categories: U14 (13.15 ± 0.48 years), U16 (15.02 ± 0.59 years), and U18 (16.24 ± 0.21). The sample consisted of kayakers (59 U14, 73 U16, and 17 U18) who attended the training camps of the respective national teams and those who did not. All participants had at least 1 year of paddling experience and had participated in national competitions. The exclusion criteria were mandatory approval of the required medical exams, non-smoking status, and no history of alcohol consumption. Written informed consent from parents or guardians was obtained.

### 2.2 Anthropometry

One certified level 3 International Society for the Advancement of Kinanthropometry (ISAK) evaluator took all anthropometric measures following the protocols established by ISAK (Esparza-Ros et al., 2019). Body mass (kg) was determined using a SECA 878 (Digital scale, SECA, Germany). Stretch stature (cm) and sitting height (cm) were determined with a SECA 206 (Portable stadiometer, SECA, Germany). Arm span (cm) was measured using a metallic tape (Stanley, United States); the arm length (cm) and forearm length (cm) were measured using a sliding calliper (Siber-Hegner GPM Calliper, Switzerland). The biacromial diameter (cm) was measured using a sliding anthropometer (Siber-Hegner GPM Anthropometer, Switzerland), and the chest girth (cm) was measured using an anthropometric tape (Lufkin W606PM, United States). All instruments were verified and calibrated before each testing session to avoid measurement errors. All measurements were taken two or three times, depending on whether the difference between the first two measurements was greater than 1%. The mean or median of the two or three measurements was used, respectively.

### 2.3 Maturity status

Somatic maturation was used to evaluate biological development (Baxter-Jones et al., 2005) by assessing the maturity offset (Mirwald et al., 2002). This method is an indicator of the temporal distribution and proposes estimating the distance in years during which the subject is at peak height velocity (PHV). This value can be negative (if the PHV has not yet been reached) or positive (if the PHV has already been exceeded). Also, the percentage of predicted adult height (PAH%) defined the maturity status, and the athlete-predicted adult height (PAH) was estimated using the Sherar et al. (2005) method.

### 2.4 Equipment setup

**FIGURE 1:**
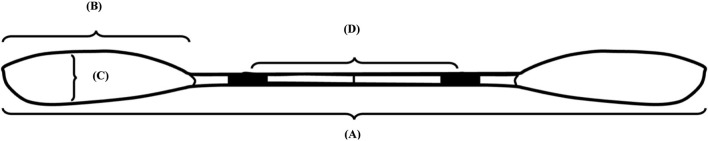
Schematic diagram of equipment setup dimensions: **(A)** Paddle length; **(B)** Blade length; **(C)** Blade width, and **(D)** Handgrip distance.

The paddle setup of the athletes (Figure 1) was measured by the same investigator for paddle and blade length and width, and handgrip distance (Ong et al., 2005). The paddle length (cm), the horizontal distance between the tips of the blades, was measured using metallic tape (Stanley, United States). Blade length (cm), the horizontal distance between the tip of the blade and the point of the shaft where the structure begins to form the blade, blade width (cm), the maximum width of the blade, and handgrip distance (cm), the horizontal distance between the joints of the third digit with the athlete using the usual grip on the paddle shaft, were measured using a sliding calliper (Siber-Hegner GPM Anthropometer, Switzerland). All measurements were taken twice, and the mean of the two measurements was used.

### 2.5 Statistical analysis

The normality and homogeneity of the variance hypotheses were verified using the Kolmogorov-Smirnov and Levene tests. Descriptive statistics were processed for each of the variables. Group mean comparisons for maturity, anthropometry, and paddle setup were assessed by the Kruskal-Wallis test, and *post hoc* tests with Bonferroni corrections were applied. Spearman’s correlation coefficients (*r*_*s*_) were calculated to examine the correlation between paddle set-up and maturational and anthropometric characteristics. These coefficients were considered as a very high correlation when the value is between 0.9 and 1, a high correlation when between 0.7 and 0.9, a moderate correlation when between 0.5 and 0.7, a low correlation when between 0.3 and 0.5, and a negligible correlation when between 0 and 0.3 (Mukaka, 2012). Cohen’s *f* ^2^ was used to measure the effect size of the observed differences and was considered small when the value was between 0.02 and 0.15, moderate when between 0.15 and 0.35, and large when the effect was >0.35 (Cohen, 1988). A 95% confidence interval (95% CI) was also calculated.

Additionally, a stepwise multiple linear regression analysis was performed to determine which anthropometric and maturity attributes could predict the configuration of the paddle and blade length, blade width, and handgrip distance for the total sample and each category analysed. The stepwise method was selected because it allows the removal of a variable whose importance in the model is reduced by adding new variables. The threshold value for inclusion was set at 0.05 and for removal at 0.1. The first variable computed was chronological age, followed by the maturation-related variables in the order described: %PAH and maturity offset. The anthropometric variables were then added subsequently in the following order: stretcth stature, body mass, wingspan, sitting height, arm length, forearm length, biacromial diameter, and chest girth. The final variables incorporated into the model were those related to paddle setup, in the specified order: paddle width, blade length, and handgrip distance. Only variables with at least a moderate correlation were included in the stepwise regression analysis. To avoid multicollinearity, predictor variables with variance inflation factor values greater than 10 and/or tolerance levels below 0.1 were excluded from the model (Marcoulides and Raykov, 2019). Because the study uses previously collected data, it was not possible to perform an *a priori* sample-size calculation. However, we included the number of participants in each subgroup and conducted a *post hoc* analysis using the observed *R* ^2^ values. This showed that the total-sample model had strong explanatory capacity (*R* ^2^ = 0.752), while the subgroup models ranged from moderate (U16, *R* ^2^ = 0.333) to strong (U14 and U18). These results indicate that the total-sample model is adequately supported by the available data, whereas subgroup analyses should be interpreted with caution.

The level of significance was set at *p* < 0.05. Statistical analyses were performed using SPSS Statistics 27.0 (SPSS Inc., Chicago, IL, United States).

## 3 Results

The mean values for maturity, anthropometry, and paddle setup parameters for the total sample and kayakers grouped by age are presented in Tables 1,2, respectively. Only PAH (*r*_*s*_ = 0.002) does not correlate significantly with paddle set-up measures in the total sample (Table 1).

**TABLE 1:** Mean (±SD) and 95% Confidence Interval (95% CI) for CA, maturity, anthropometry, paddle setup, and its correlation (*rs*) with paddle length, blade length, blade width, and handgrip distance for the total sample.

Variables	Kayakers total sample (n = 149)					
	Mean (±SD)	95% CI	Paddle length	Blade length	Blade width	Handgrip distance
CA (years)	14.42 ± 1.20	12.05–16.79	0.723**	0.562**	0.575**	0.454**
Maturity						
Maturity offset (years)	0.98 ± 1.18	−1.35–3.32	0.789**	0.603**	0.614**	0.465**
PAH (cm)	180.19 ± 4.93	170.52–189.87	0.002	−0.103	−0.146	0.019
PAH (%)	94.35 ± 4.30	85.91–102.79	0.792**	0.600**	0.607**	0.425**
Anthropometry						
Body mass (kg)	62.04 ± 11.03	40.41–83.68	0.628**	0.543**	0.567**	0.422**
Stretch stature (cm)	169.98 ± 8.24	153.82–186.14	0.712**	0.512**	0.487**	0.457**
Sitting height (cm)	89.10 ± 4.87	79.55–98.65	0.691**	0.493**	0.509**	0.420**
Arm span (cm)	174.18 ± 9.80	154.97–193.41	0.651**	0.498**	0.438**	0.492**
Arm length (cm)	31.92 ± 2.45	27.10–36.74	0.685**	0.436**	0.418**	0.409**
Forearm length (cm)	25.33 ± 2.21	20.99–29.67	0.544**	0.264**	0.324**	0.380**
Chest girth (cm)	89.55 ± 7.65	74.55–104.56	0.641**	0.550**	0.597**	0.409**
Biacromial diameter (cm)	37.85 ± 3.64	29.39–45.72	0.610**	0.596**	0.564**	0.430**
Paddle setup						
Paddle length (cm)	209.27 ± 6.86	195.83–222.73	−	0.658**	0.627**	0.511**
Blade length (cm)	47.56 ± 4.25	43.87–52.15	0.627**	−	0.789**	0.245**
Blade width (cm)	16.03 ± 3.95	14.20–16.98	0.658**	0.789**	−	0.204*
Handgrip distance (cm)	69.73 ± 5.44	59.06–80.40	0.511**	0.245**	0.204*	−

CA, chronological age; PAH, predicted adult height. *Correlation is significant at the level *p* < 0.05. ** Correlation is significant at the level *p* < 0.01.

**TABLE 2:** Mean (±SD) and 95% Confidence Interval (95% CI) for CA, maturity, anthropometry, paddle setup and its correlation (*rs*) with paddle length, blade length, blade width and handgrip distance, for kayakers grouped by age.

Variables	Kayakers grouped by age (n = 149) - mean (±SD)																	
	U14 (n = 59)						U16 (n = 73)						U18 (n = 17)					
	Mean ± SD	95% CI	Paddle length	Blade length	Blade width	Handgrip distance	Mean ± SD	95% CI	Paddle length	Blade length	Blade width	Handgrip distance	Mean ± SD	95% CI	Paddle length	Blade length	Blade width	Handgrip distance
CA (years)	13.15 ± 0.48	12.21–14.10	0.436**	0.155	0.236	0.411**	15.02 ± 0.59^∂^	13.86–16.19	0.342**	−0.041	−0.012	0.086	16.24 ± 0.21^†§^	15.82–16.66	0.331	0.402	0.187	0.402
Maturity																		
Maturity offset (years)	−0.17 ± 0.81	−1.77–1.42	0.732**	0.334**	0.380**	0.603**	1.58 ± 0.59^∂^	0.41–2.76	0.502**	0.139	0.142	0.002	2.41 ± 0.51^†§^	1.41–3.42	0.343	0.296	−0.067	0.486*
PAH (cm)	181.84 ± 4.45	173.11–190.59	0.356*	0.106	0.206	0.262*	179.22 ± 5.32^∂^	168.78–189.66	0.211	0.078	−0.094	0.053	178.66 ± 2.97^†^	172.83–184.50	0.328	0.013	−0.029	0.488
PAH (%)	90.03 ± 3.41	83.34–96.73	0.733**	0.290*	0.372**	0.602**	96.84 ± 1.61^∂^	93.69–100.00	0.513**	0.114	0.085	−0.127	98.62 ± 1.04^†§^	96.57–100.68	0.443	0.349	−0.025	0.419
Anthropometry																		
Body Mass (kg)	54.43 ± 9.74	35.34–73.52	0.687**	0.351**	0.335**	0.528**	66.14 ± 8.55^∂^	49.38–82.91	0.222	0.329**	0.367**	−0.056	70.85 ± 9.00^†^	53.21–88.49	0.486*	0.413	0.157	0.629**
Stretch stature (cm)	163.76 ± 8.01	148.06–179.47	0.734**	0.314*	0.412**	0.599**	173.55 ± 5.56^∂^	162.65–184.46	0.433**	0.182	−0.001	−0.011	176.21 ± 3.78^†^	168.79–183.63	0.488*	0.280	0.079	0.377
Sitting height (cm)	85.56 ± 5.02	75.72–95.41	0.739**	0.351**	0.389**	0.597**	91.22 ± 3.01^∂^	85.30–97.14	0.439**	0.124	0.112	−0.023	92.25 ± 3.16^†^	86.06–98.46	0.181	0.125	−0.136	0.249
Arm span (cm)	167.02 ± 9.68	148.05–186.01	0.608**	0.311*	0.325*	0.524**	178.35 ± 6.48^∂^	165.65–191.06	0.347**	0.173	−0.039	0.122	181.13 ± 6.85^†^	168.58–193.70	0.624**	0.237	0.103	0.584*
Arm length (cm)	30.02 ± 2.00	26.10–33.94	0.602**	0.345**	0.291*	0.362**	33.05 ± 1.85^∂^	29.43–36.69	0.432**	−0.084	−0.122	0.083	33.65 ± 1.87^†^	29.98–37.34	0.492*	0.060	−0.096	0.291
Forearm length (cm)	23.67 ± 1.71	20.32–27.04	0.389**	−0.054	0.110	0.442**	26.43 ± 1.92^∂^	22.66–30.20	0.190	−0.254*	−0.146	−0.013	26.35 ± 1.13^†^	24.12–28.58	0.398	0.016	−0.215	0.308
Chest girth (cm)	83.20 ± 5.79	71.84–94.56	0.595**	0.216	0.262*	0.385**	92.82 ± 5.31^∂^	82.41–103.24	0.208	0.279*	0.352**	−0.024	97.58 ± 5.17^†^	87.44–107.74	0.197	0.244	0.112	0.455
Biacromial diameter (cm)	36.14 ± 2.58	31.09–41.21	0.637**	0.391**	0.340**	0.510**	39.16 ± 1.59^∂^	36.04–42.29	0.154	0.323**	0.257*	−0.089	39.95 ± 2.31^†^	35.43–44.49	0.655**	0.458	0.340	0.463
Paddle setup																		
Paddle length (cm)	203.95 ± 7.40	189.45–218.46	−	0.543**	0.549**	0.621**	212.22 ± 3.34^∂^	205.68–218.77	−	0.328**	0.170	0.114	215.11 ± 2.52^†§^	210.16–220.07	−	0.652**	0.315	0.501*
Blade length (cm)	46.55 ± 2.22	42.19–50.92	0.543**	−	0.696**	0.231	48.84 ± 1.46^∂^	45.96–51.72	0.328**	−	0.517**	−0.180	49.48 ± 0.57^†^	48.36–50.61	0.652**	−	0.706**	0.310
Blade width (cm)	15.07 ± 0.63	13.83–16.32	0.549**	0.696**	−	0.191	15.87 ± 0.53^∂^	14.82–16.92	0.170	0.517**	−	−0.267*	16.19 ± 0.39^†^	15.41–16.97	0.315	0.706**	−	−0.003
Handgrip distance (cm)	67.09 ± 6.07	55.19–79.00	0.621**	0.231	0.191	−	70.87 ± 3.90^∂^	63.22–78.53	0.114	−0.180	−0.267*	−	73.96 ± 4.59^†^	64.95–82.98	0.501*	0.310	−0.003	−

CA, chronological age; PAH, predicted adult height. *Correlation is significant at level *p* < 0.05. ** Correlation is significant at level *p* < 0.01; ^∂^Significant difference (p < 0.05) between U14 and U16; ^†^Significant difference (p < 0.05) between U14 and U18 and ^§^Significant difference (p < 0.05) between U16 and U18.

For kayakers grouped by age, significant differences (*p* < 0.05) were observed in CA and all maturity and anthropometric parameters between U14 and U16, and between U14 and U18. Between U16 and U18, significant differences (*p* < 0.05) were observed only in CA, maturity offset, PAH%, and paddle length (Table 2).

The stepwise linear regression equations are shown in Table 3 for the total sample and Table 4 for kayakers grouped by age. In the total sample, PAH% and stretch stature significantly contributed to the prediction of paddle length (*p* < 0.01). In U14, paddle length was also significantly predicted by PAH% (*p* < 0.01) and stretch stature (*p* < 0.05), while in U16 it was significantly predicted by maturity offset and %PAH (*p* < 0.05), and in U18 by biacromial diameter (*p* < 0.01).

**TABLE 3:** Regression equations for the total sample to predict paddle and blade length and blade width for the total sample.

Variable	Total Sample	*R* ^2^	Adjusted R^2^	SEE (cm)	VIF	Tolerance levels	*f * ^2^
Paddle length	Paddle Length = 76.416 + (0.934 × %PAH**) + (0.263 × stretch stature**)	0.752	0.749	3.439	3.263	0.263	1.74
Blade length	Blade Length = 20.567 + (0.214 × %PAH**) + (0.191 × biacromial diameter*)	0.408	0.400	1.636	2.578	0.388	0.83
Blade width	Blade Width = 6.563 + (0.073 × %PAH**) + (0.024 × chest girth*)	0.445	0.437	0.531	2.661	0.376	0.90

%PAH: percentage of predicted adult height; VIF: variation inflation factor; *f* ^2^: Cohen effect size.

*Significant contribution (*p* < 0.05) to the predictive model; **Significant contribution (*p* < 0.01) to the predictive model.

**TABLE 4:** Regression equations for the categories U14 to predict the paddle length and the handgrip distance, U16 to predict the paddle length, and U18 to predict the paddle length and the handgrip distance.

Variable	U14	*R* ^2^	Adjusted R^2^	SEE (cm)	VIF	Tolerance levels	*f * ^2^
Paddle length	Paddle Length = 55.719 + (0.972 × %PAH**) + (0.371 × stretch stature*)	0.677	0.666	4.280	4.212	0.237	1.45
Handgrip distance	Handgrip distance = − 18.896 + (0.525 × stretch stature**)	0.480	0.471	4.417	1.000	1.000	0.96
	U16						
Paddle length	Paddle Length = 150.344 + (1.813 × maturity offset*) + (0.609 × %PAH*)	0.333	0.314	2.767	2.174	0.460	0.71
	U18						
Paddle length	Paddle Length = 182.773 + (0.809 × biacromial diameter**)	0.547	0.517	1.756	1.000	1.000	1.10
Handgrip distance	Handgrip distance = 53.718 + (0.286 × body Mass*)	0.313	0.267	3.936	1.000	1.000	0.67

%PAH: percentage of predicted adult height; VIF: variation inflation factor; *f* ^2^: Cohen effect size.

*Significant contribution (*p* < 0.05) to the predictive model; **Significant contribution (*p* < 0.01) to the predictive model.

**FIGURE 2:**
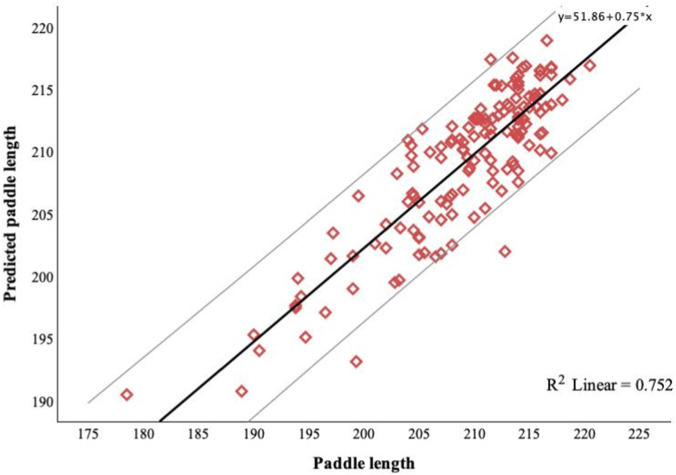
Linear relationship between predicted paddle length and observed paddle length with 95% Confidence Interval lines for the total sample.

The predictive equation for the total sample represented 75% of the variance in paddle length (*b* = 76.416, 95% CI [63.931, 88.902], *p* < 0.001). At the same time, the predictive equations for U14, U16, and U18 accounted for 67%, 33%, and 54% of the variance in paddle length, respectively. Figure 2 shows the linear relationship between predicted paddle length and observed paddle length with 95% Confidence Interval lines for the total sample. A *post hoc* evaluation of model strength based on *R* ^2^ confirmed that the total-sample model (*R* ^2^ = 0.75) is robust, while the subgroup models showed lower explanatory power, particularly U16 (*R* ^2^ = 0.33).

## 4 Discussion

This study aimed to develop predictive equations to help determine optimal paddle scaling for kayakers competing in three age categories, U14, U16, and U18. The main contribution of this study was the development of predictive equations for different paddle fittings. Considering the total sample, one equation could explain 75% of the variation in paddle length. Also, when the age categories were analysed separately, the three predictive equations for U14, U16, and U18 accounted for 67, 33, and 54% of the variation in paddle length. Furthermore, the anthropometric characteristics found in this study for U14 and U16 kayakers were similar to previous studies (Fernandes et al., 2021) and seem to move parallel to the paddle setup characteristics of the three evaluated groups.

An interesting finding was that the younger the age group, the more positively correlated the anthropometric and maturity variables were with the paddle length. This finding may suggest that anthropometric and maturity characteristics have a greater impact on equipment selection at younger ages. Implying that, as the athlete ages, other factors (i.e., strength, paddling style, technical efficiency, etc.) may condition the choice of paddle setup. Recently, Fernandes et al. (2021) showed that years of practice are crucial in youth kayaking performances. Likewise, Kristiansen et al. (2023) reported that upper-body strength is vital for performance in sprint kayaking. This fact appears to align with Cox (1992), who stated that achieving a sprint paddler’s maximum possible performance is a lengthy process influenced by many interrelated factors. For example, improved fitness will likely enhance technique. Although it may be possible to have a good, efficient paddling technique without the physical fitness to sustain it, it is also challenging to have a proper technique with poorly designed or inadequate equipment. This suggestion may be corroborated by the U16, low paddle length predictor equation of 33%, and may be associated with the marked gain of 12 kg in body mass from the U14 (54.43 ± 9.74 kg) to the U16 (66.14 ± 8.55 kg) category. This mass gain may consequently affect strength, which can have direct consequences on the appropriate selection of the correct paddle size, forcing coaches to try out various setup alternatives in a short period of time, not allowing the young kayaker the necessary time to adapt to the new equipment and with that, possibly impairing the ability to achieve technical efficiency, and probably being a sensitive period in the development of aspects related to technique.

In the present study, the anthropometric characteristics that showed a higher correlation with paddle length were stretch stature and sitting height for the total sample, as well as for the U14 and U16 categories (*p* < 0.01). Additionally, the biacromial diameter and arm span of the U18 category showed a significant correlation (*p* < 0.01). Regarding maturity status, maturity offset, and %PAH, significant correlations (p < 0.01) were observed with paddle length for the total sample, U14, and U16. In a previous study, Alacid et al. (2014) showed that paddle length correlated strongly with stretch stature.

In the study of Ong et al. (2005), stature was the anthropometric characteristic most associated with the equipment setup for elite male sprint kayakers, serving as a predictor of hand grip distance (*R* ^*2*^ = 0.541; *p* < 0.001) and foot bar distance (*R* ^*2*^ = 0.589; *p* < 0.001), the latter being a variable related to kayak fitting. These authors stated that regression analyses revealed significant relationships among measures of body size, paddle length, and blade length. However, only 20% and 25% of the variance in the dependent variables was accounted for. Diafas et al. (2012) reported that total arm length, arm span, total leg length, stature (*r* = 0.33, p < 0.01), body mass, and lean body fat (*r* = 0.44, *p <* 0.001) were significantly correlated with paddle length. Moreover, a study by Ong et al. (2006), presented the paddle length as 121.4% of the stretch stature in elite kayakers, and Alacid et al. (2014), studying young kayakers, presented it as 121.9%. In the present study, the results were similar, with the paddle length expressing 123.1% of the stretch stature and 120.2% of the arm span in the total sample.

This study is not without limitations. The *post hoc* evaluation suggests that, although the available data well support the total-sample equation, the subgroup equations—particularly U16—may be influenced by smaller sample sizes and greater within-group variability. In the U16 category, this lower explanatory power may also be related to the nonlinear growth and maturation changes typical of mid-adolescence, which can reduce the stability of linear models. For this reason, the subgroup results should be viewed as exploratory and interpreted with caution.

Additionally, the sample is not homogeneous across the evaluated categories, and the lack of data on the athletes’ physical fitness and performance may also be a limitation. An experimental study is recommended to verify the predictive equations derived in this study, evaluate the effect of technique, and assess the reliability of using predictive models to select a paddle setup for young kayakers. The use of data collected between 2005 and 2018 is also a limitation of this study, as changes in investigators’ training or equipment calibration over time may have introduced inconsistencies.

In conclusion, from the several predictive equations developed to help scale the setup of the paddle of the young kayakers, the one that best explains the variation in paddle length is the one developed for the total sample (*R* ^*2*^ = 0.75; *SEE* = 3.43, *p* < 0.01) and goes as follows:

$$\left[\text{Paddle length}=76.416+\left(0.934\times \%{\text{PAH}}^{**}\right)+\left(0.263\times {\text{Stretch Stature}}^{**}\right)\right].$$

These findings may prove extremely important, as they explain the variance in paddle length in 75% of cases and can be used by coaches and kayakers as a more objective guide for the initial setup of their paddle length, thereby avoiding the traditional empirical method described in the introduction. Furthermore, it is essential to mention that, given 25% of the paddle length variation may be due to several other factors, the predictive equations derived in this study should be regarded as initial guidelines rather than a definitive prescription. Validation in independent or more diverse samples is therefore also recommended.

## Data Availability

The raw data supporting the conclusions of this article will be made available by the authors, without undue reservation.
